# Recent advances in aptamer-based targeted drug delivery systems for cancer therapy

**DOI:** 10.3389/fbioe.2022.972933

**Published:** 2022-08-16

**Authors:** Fei Gao, Jianhui Yin, Yan Chen, Changyong Guo, Honggang Hu, Jiacan Su

**Affiliations:** ^1^ Institude of Translation Medicine, Shanghai University, Shanghai, China; ^2^ Department of Pharmacy, Medical Supplies Center of PLA General Hospital, Beijing, China

**Keywords:** aptamers, targeted drug delivery, cancer therapy, aptamer-drug conjugates (ApDCs), aptamer-based nanomaterial system

## Abstract

The past decade has become an important strategy in precision medicine for the targeted therapy of many diseases, expecially various types of cancer. As a promising targeted element, nucleic acid aptamers are single-stranded functional oligonucleotides which have specific abilities to bind with various target molecules ranging from small molecules to entire organisms. They are often named ‘chemical antibody’ and have aroused extensive interest in diverse clinical studies on account of their advantages, such as considerable biostability, versatile chemical modification, low immunogenicity and quick tissue penetration. Thus, aptamer-embedded drug delivery systems offer an unprecedented opportunity in bioanalysis and biomedicine. In this short review, we endeavor to discuss the recent advances in aptamer-based targeted drug delivery platforms for cancer therapy. Some perspectives on the advantages, challenges and opportunities are also presented.

## 1 Introduction

Aptamers are a special class of DNA or RNA oligonucleotides that fold up into unique three-dimensional (3D) conformations for specifically recognizing cognate molecular targets (Miao et al., 2021). Aptamers are usually screened via an *in vitro* iterative method named Systematic Evolution of Ligands by EXponential Enrichment (SELEX) (Tan et al., 2013), which was independently discovered by two American groups in early 1990s (Ni et al., 2011). In recent years, various aptamers have been isolated for diverse types of target molecules (Table 1), including organic and inorganic molecules, peptides, proteins, nucleic acids, bacterium and even live cells, such as EpCAM aptamer binds to epithelial cell adhesion molecules (EpCAM) and aptamer sgc8 against protein tyrosine kinase-7 (PTK-7) (Ni et al., 2021). Compared with other targeted ligands, aptamers possess many excellent properties, including high chemical stability and binding affinity, versatile chemical modification, low or even non immunogenicity, small size and quick tissue penetration (Yang et al., 2022). These remarkable advantages make aptamers widely used in the field of cancer targeted therapy (Sun et al., 2022) and diagnosis (Zhang et al., 2020). This review will predominantly provide a brief overview of recent researches on aptamer-based targeted systems for cancer therapy. The future possibilities and challenges of aptamer guided drug delivery system will also be discussed.

**TABLE 1:** Examples of therapeutic aptamers in clinical stages for cancer therapy.

Molecular targets	Names of aptamer examples (Blank means no self-explanation)	Disease indication	References
HER2	Herceptamers	Cancer	(Chi-hong et al., 2003; Thiel et al., 2012; Varmira et al., 2013; Varmira et al., 2014; Zhu et al., 2017)
EGFR	E07	Cancer	(Esposito et al., 2011; Li et al., 2011; Wang et al., 2014a)
EpCAM	SYL3C, Ep1	Cancer	(Song et al., 2013; Xiang et al., 2015)
VEGF	Pegaptanib (PEGylated), VEap121	AMD, Cancer	Gragoudas et al. (2004)
Nucleolin	AS1411	Cancer	(Ireson and Kelland, 2006; Bates et al., 2009; Li et al., 2017; Liang et al., 2017; Weng et al., 2018; Fu and Xiang, 2020; Vandghanooni et al., 2020; He et al., 2021)
PTK7	sgc8	Cancer	(Shangguan et al., 2008; Huang et al., 2009; Wang et al., 2014b; Yang et al., 2015b; Cao et al., 2017; Gong et al., 2020)
IGHM	Td05	Cancer	(Mallikaratchy et al., 2007; Yang et al., 2015b)
αvβ3 integrin	Apt-αvβ3	Cancer	Mi et al. (2005)
NF-κB	Y1, Y3	Cancer	Lebruska and Maher, (1999)
E2F3 transcription factor	aptamer 8–2	Cancer	(Ishizaki et al., 1996; Martell et al., 2002)
HER3	A30	Cancer	Chen et al. (2003)
CD30	C2, NGS6.0	Cancer	Zhang et al. (2009)
CTLA-4	CTLA4^apt^, aptCTLA-4	Cancer	(Herrmann et al., 2014; Huang et al., 2017)
OX40	9C7, 11F11, 9D9	Immune diseases, including Cancer	(Pratico et al., 2013; Soldevilla et al., 2015)
PD-1	MP7	Immune diseases, including Cancer	Prodeus et al. (2015)
PD-L1	aptPD-L1	Immune diseases, including Cancer	Lai et al. (2016)
Tim-3	TIM3Apt	Immune diseases, including Cancer	Gefen et al. (2017)
LAG3	Apt1, Apt2, Apt4, Apt5	Immune diseases, including Cancer	Soldevilla et al. (2017)
CD28	AptCD28	Immune diseases, including Cancer	Lozano et al. (2016)
DEC205		Immune diseases, including Cancer	Wengerter et al. (2014)
IL-4Ra	cl.42	Immune diseases, including Cancer	Liu et al. (2017)
IL-6R	AIR-3	Immune diseases, including Cancer	Kruspe et al. (2014)
PLK1, BLC2		Cancer	McNamara et al. (2006)
Mucin-1, BCL2		Cancer	Jeong et al. (2017)
polynucleotide		Cancer	Nooranian et al. (2021)
Mucin1	AptA, AptB, S2.2	Cancer	(Elghanian et al., 1997; Ferreira et al., 2006; Zhang et al., 2014b; Hu et al., 2014)
OS cell	LC09	Cancer	Zhao et al. (2019)
Adenosine		Cancer	Li et al. (2018a)
ALPL protein	Apt19S	Cancer	Xuan et al. (2020)
PSMA	A9, A10	Cancer	Lupold et al. (2002)

HER: human epidermal growth factor receptor. EGFR: epidermal growth factor receptor. AMD: age-related macular degeneration. EpCAM: epithelial cell adhesion molecule. VEGF: vascular endothelial growth factor. PSMA: prostate-specific membrane antigen. PTK7: protein tyrosine kinase 7. IGHM: immunoglobulin *μ* heavy chains. CTLA-4: cytotoxic T-lymphocyte associated protein 4. PD-1: programmed death receptor I. PD-L1: programmed death ligand I. Tim-3: T cell immunoglobulin-3. LAG3: lymphocyte-activation gene 3. IL-6R: interleukin 6 receptor. PLK1: polo-like kinase 1. BLC2: B-cell lymphoma.

## 2 Aptamers as therapeutic agents

Aptamers, as therapeutic agents, can effectively recognize various proteins on the cell membrane or in the blood circulation to modulate their interaction with receptors and affect the corresponding biological pathways for the treatment of various diseases (Zhou and Rossi, 2017). The ongoing progresses in biomedical technology are encouraging the development of aptamers as therapeutic agents for improving human health. Over the past few decades, the number of therapeutic aptamers in clinical stages has been increasing (Nimjee1 et al., 2017). In 2004, Pegaptanib (Macugen), as the first aptamer in clinical use, was approved by the FDA to treat Age-related macular degeneration (AMD), which was known to the leading reason of blindness in many aging people (Ng et al., 2006). Vascular endothelial growth factor (VEGF) can increase vascular permeability and induce angiogenesis, leading to AMD (Ucuzian et al., 2010). Pegaptanib as an anti-VEGF antagonist aptamer can specifically block VEGF and interfere with the interaction of VEGF and its receptors to treat AMD. For increasing its *in vivo* biostability, 40 kDa monomethoxypolyethylene glycol (PEG) was then conjugated with pegatanib to decrease nuclease degradation. However, the antibody fragment ranibizumab (Lucentis; Genentech) has recently occupied significant market due to blocking all types of human VEGF even and the smallest VEGF121 (Martin et al., 2011).

Another famous therapeutic aptamer AS1411 has been in clinical phase II trial for the treatment of metastatic renal cell carcinoma. AS1411 composed of thymine and guanines can form special guanine-mediated quadraplex structures in solution (Ireson and Kelland, 2006). Due to this unique three-dimensional (3D) structures, AS1411 can target to nucleolin protein with high specificity, which was normally found overexpressing on the surface of cancer cells. Unlike other aptamers, AS1411 was discovered by screening antisense oligonucleotides for antiproliferation effect (Bates et al., 1999). Although the underlying mechanisms of AS1411-based antiproliferation effect have not been fully comprehended, it has showed growth-antitumor abilities against a widely range of tumor cells through multiple signaling pathways involving *BCL-2* mRNA destabilization and NF-κB inhibition (Soundararajan et al., 2008). Some studies have proved that guanine deaminase is an important pathway in affecting the cell-type selectivity to the anti-proliferation function of guanine-based biomolecules (Wang et al., 2019). So this rich guanine aptamer, as one of the most promising aptamers, has great hope to be used in clinical cancer therapy owing to its outstanding safety profile and anticancer ability in some intractable tumors (Zhang et al., 2015). In addition, there were some studies about AS1411 derivatives which were obtained by chemical modification with alternative nucleobases or backbones for improving chemical and biological properties. In 2016, Fan *et al.* reported the first AS1411 derivative that showed excellent ability in the inhibition of DNA replication and tumor cell growth, and induced S-phase cell cycle arrest via chemical modification of 2′-deoxyinosine in AS1411 aptamer (Cai et al., 2014). Subsequently, they also developed another strategy to modify AS1411 aptamer through the use of 2′-deoxyinosine (2′-dI) and D-/l-isothymidine (D-/L-isoT) for improving the bioactivity of AS1411 aptamer (Fan, Sun, Wu, Zhang, Yang). In addition to exploring aptamers for directly inhibiting cancer cells growth, aptamers can also indirectly display anticancer abilities through modulating the immune system. In recent years, some agonistic aptamers with immunomodulatory properties have been found. It is worth noting that these aptamers recognizing 4-1BB or OX40 almost show similar or even superior immunomodulatory ability to the corresponding antibodies, followed with similar anticancer effects. Examples of this type of aptamers include multimeric aptamers that can target 4-1BB (CD137) on activated T cells and improve T cell proliferation, IL-2 secretion, survival and cytolytic activity of T cells (McNamara et al., 2008).

## 3 Aptamer-drug conjugates for targeted drug delivery

In addition to being therapeutic agents, aptamers have been more widely explored as targeting carriers for the therapeutic agents delivery, such as chemotherapeutics, small interfering RNAs (siRNAs), microRNAs (miRNAs), toxins and so on (Xuan et al., 2018). Traditional ApDCs are mostly comprised of aptamers attached to various potent drugs through all kinds of cleavable or non-cleavable linkers. Compared with antibody-drug conjugates (ADCs), a few of which have been applied to clinical treatment of cancer, ApDCs show many significant advantages, including relatively small size, synthesis procedure economy, chemical modification simplicity and tissue penetration speedy (Chen et al., 2017). Based on diseased-related biomarkers, ApDCs have been developed for a wide range of therapeutic modalities, such as chemotherapy, immunotherapy and so on.

Chemotherapy is one of the most fashional therapeutic modalities for cancer, but this conventional strategy suffers from serious drug toxicity in healthy tissues and various side effects. For improving therapeutic efficacy and diminishing side effects, ApDC-mediated targeted drug delivery has been studied. As an example, Tan *et al.* designed and synthesized a sgc8-Dox conjugate for targeted delivery of doxorubicin (Dox) (Huang et al., 2009). In this ApDCs, antitumor agent Dox was conjugated with aptamer sgc8 at a 1:1 ratio via an acid-labile hydrazone linker, such that Dox can be selectively delivered in acidic tumor environment. Zhang *et al.* reported a water-soluble nucleolin aptamer-paclitaxel conjugate that can specifically release PTX to the tumor site via a cathepsin B-labile valine-citrulline dipeptide linker (Li et al., 2017). Recently, a nucleolin aptamer (AS1411) loaded with BET-targeting PROTAC against breast cancer stem cells was reported by Sheng *et al* (He et al., 2021)*.* Notably, the aptamer/drug ratio is essential in achieving excellent therapeutic efficacy. For maximizing drug delivery efficiency, a phosphoramidite prodrug of 5-fluorouracil (5-FU) was developed and the resulting ApDCs with multiple drug copies can be synthesized via automated nucleic acid synthesis using standard solid-phase DNA synthesis chemistry (Wang et al., 2014b). In order to control drug release, a photocleavable linker was added to the bone of phosphoramidite prodrug. As a result, the ApDCs not only were efficiently internalized into cancer cells, but also showed specific drug release in a photocontrollable manner.

Besides conjugating with chemotherapeutic drugs, aptamer can also link with therapeutic RNA or DNA. In recent years, gene therapy as a hot therapeutic modality has made great breakthrough in the treatment of cancer (Song et al., 2021). However, just like many other therapeutic drugs, most of gene therapy agents lack specific recognition ability for the disease tissues, which make it vital to specifically deliver gene therapy agents to cancer cells. As target ligands, aptamers can be utilized to improve the gene therapy safety and therapeutic efficacy (Li et al., 2013). In an early research, an ApDCs was constructed using a PSMA-targeting aptamer and a small interfering RNA (siRNA), which can silence polo-like kinase 1 (*PLK1*) and B-cell lymphoma 2 (*BLC2)* overexpressed in most human tumor cells (McNamara et al., 2006). In this study, the resulting ApDCs can not only specifically release siRNA into PSMA-positive LNCaP cells and lead to cell apoptosis, but also remarkably inhibit tumor growth in LNCaP tumor-bearing mices. Subsequently, Aptamer-siRNA conjugates have been systematically studied by PEGlation to optimize circulation half-life in the blood, by chemical modification to increase biostability, and by exploring the two-dimensional structure to improve the intracellular processing of RNA-induced genes silencing. In another research, a multiple mucin-1 aptamer was conjugated with *BCL2*-specific siRNA, and doxorubicin (Dox) was loaded into these conjugates through intercalation with nucleic acids (Jeong et al., 2017). These Dox-incorporated multivalent Apt-siRNA conjugates can overcome multidrug resistance into MDR cancer cells through aptamer-mediated codelivery of Dox and siRNA. Note that the 3D structure of multivalent Dox-Apt-siRNA were well defined, which is beneficial for their clinical application. Furthermore, aptamers were also successfully conjugated with other nucleic acid gene therapeutics, such as small hairpin RNA (shRNA) and microRNA (miRNA) (Soldevilla et al., 2018).

## 4 Aptamer-based nanomaterial system for targeted drug delivery

**FIGURE 1:**
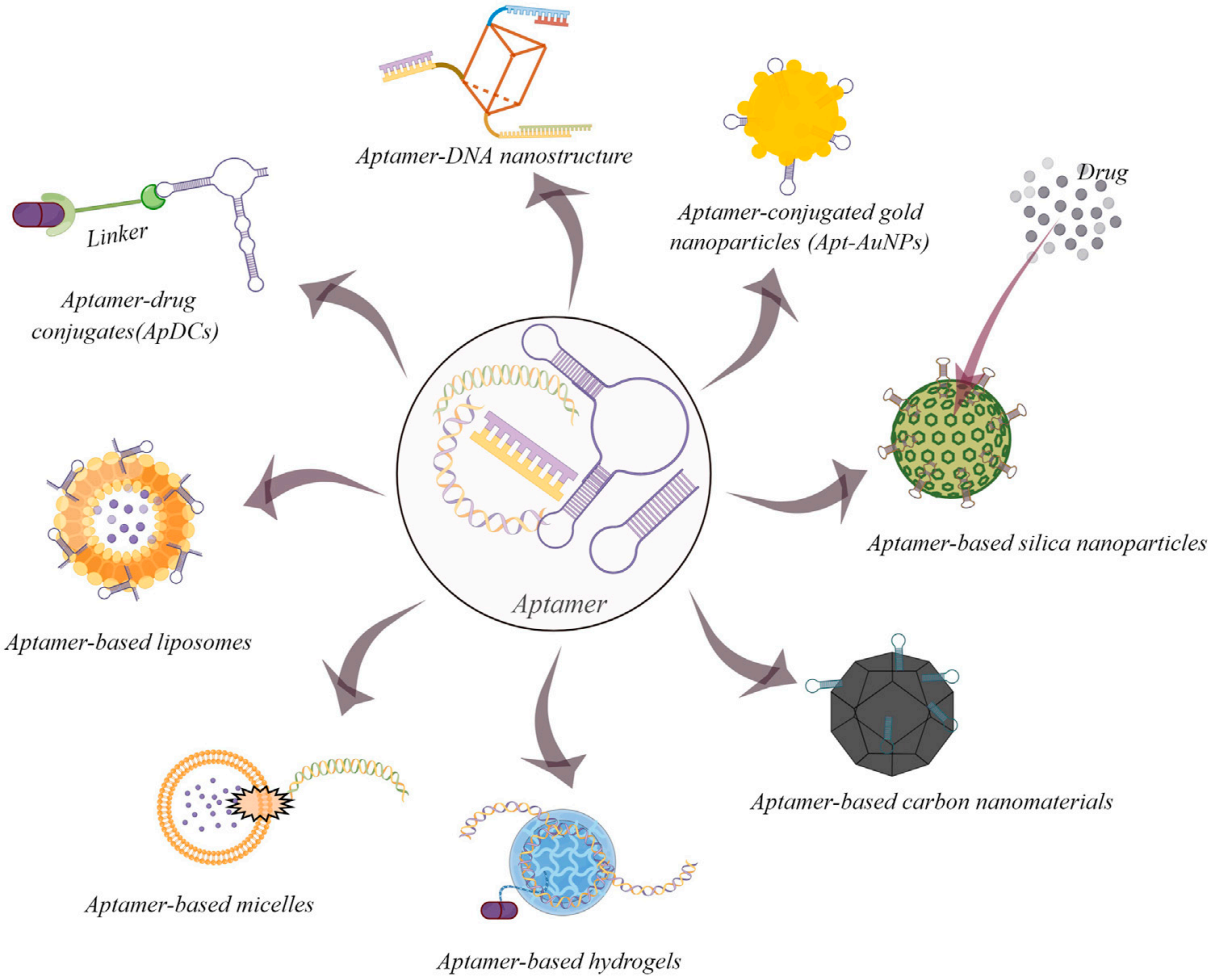
Some common examples of aptamer-based drug delivery systems for cancer therapy (By figdraw).

Nanomaterials play a crucial role in the application of bioanalysis and biomedicine (Li et al., 2021). Due to their unique physicochemical properties, including an ultra-small size, a large surface area and loading ability, nanomaterials have overcome many limitations of conventional therapeutic and diagnostic strategies (Dong et al., 2020). The key of nanomedicine development is to improve the specific recognition ability for disease tissues (Liu et al., 2021). The combination of aptamers and nanomaterials is a promising progress for targeted drug delivery (Figure 1) (Mahmoudpoura et al., 2021). In this section, several representative aptamer-based inorganic and organic nanomaterials on cancer therapy would be discussed.

### 4.1 Aptamer-based inorganic nanomaterial systems

As an important inorganic nanomaterial, gold nanoparticles have gained considerable attentions in biomedicine as result of their high surface-to-volume ratio, low-toxicity, excellent stability and biological compatibility (Yang et al., 2015a). Aptamer-conjugated gold nanomaterials (Apt-AuNPs), which synergically possess special advantageous properties of aptamers and gold nanoparticles, have been widely utilized in the field of cancer diagnosis and therapy (Nooranian et al., 2021). A classical research from the Mirkin group employed target DNA molecules to form a polymeric network of nanoparticles for specifically detecting polynucleotide (Elghanian et al., 1997). Subsequently, there are emerging many corresponding studies, such as enzyme responsive Apt-AuNPs for mucin 1 protein (MUC1) detection (Hu et al., 2014), Apt-AuNPs combined with graphene oxide for the photothermal therapy of breast cancer (Yang et al., 2015b) and aptamer-functionalized AuNPs-Fe_3_O_4_-GS capture probe for monitoring circulating tumor cell in whole blood (Dou et al., 2019).

Among the set of inorganic nanomedicine, silica nanoparticles have become suitable carriers in drug delivery systems (Zou et al., 2020). These particles successfully provided controllable drug release *in vivo* and *in vitro* through the change in PH and temperature, photochemical reactions and certain redox reactions (Fu and Xiang, 2020). After combined with targeted elements such as aptamers, they can enhance cancer therapeutic effects with a lower dose of drug (Vandghanooni et al., 2020). As an early example, Cai’s group reported a novel Mesoporous Silica Nanoparticles (MSN)-based redox-responsive nanocontainer for triplex cancer targeted therapy. In their study, AS1411 aptamer was tailored onto the CytC-sealed MSNs (Zhang et al., 2014a). And this system can lead to the special release of Dox into the tumor cells via the breakage of S-S bonds. In 2021, Tan *et al.* first developed FRET-based two-photon MSNs for multiplexed intracellular imaging and targeted drug delivery (Wu et al., 2021). The MSNs can display different two-photon multicolor fluoresence by varying the doping ratio of the three dyes. Furthermore, the Dox-loaded and aptamer-capped nanosystem can be efficiently internalized into the cancer cells and release the anticancer drug Dox. In addition, aptamer-targeted MSNs have also been widely used for gene targeted delivery, which can protect gene therapy agents from degradation by nuclease (Zhang et al., 2014b).

Conventional carbon nanomaterials, including fullerene, graphene, carbon dots/nanobots/nanotubes and hybrids, exhibit unique advantages in biomedical application (Weng et al., 2018). Aptamer-functionalized carbon nanomaterials make their ideal nanoplatforms for cancer diagnostics and therapeutics (Yang et al., 2018). Recently, Wang *et al.* developed the multifunctional, which showed heat-stimulative and sustained release properties (Wang et al., 2015). With the introduction of MUC1 aptamers, this nanoparticle can detect targeted MCF-7 breast cells with excellent recognition ability. In addition, aptamer-based graphene nanomaterials have gained many fascinating developments in cancer gene therapy. In 2017, aptamer-based graphene quantum dots loaded with porphyrin derivatives photosensitizer were reported for fluorescence-guided photothermal/photodynamic synergetic therapy (Cao et al., 2017). This multifunctional theranostic nanomaterials displayed good feasibility for detecting intracellular cancer-related miRNA, whereas the intrinsic fluorescence could be used to distinguish cancer cells from somatic cells.

### 4.2 Aptamer-based organic nanomaterial systems

As the first explored drug delivery system, liposomes have many promising properties such as good biocompatibility, low toxicity, low immunogencity and excellent drug loading efficiency (Moosaviana and Sahebkar, 2019). PEGylated liposomal doxorubicin, Doxil^®^, is the first FDA-approved liposomal drug for the treatment of solid tumors. With the rapid development of biotechnology, liposomal systems with specific targeting ability have been synthesized successfully by the introduction of various molecular recognition elements, such as folate, peptides, antibodies, and aptamers. Among them, aptamer-based lipsomes have attracted widely attention (Zhao et al., 2019). In the early research, Tan *et al.* reported a therapeutic aptamer-modified liposome nanoparticle with dual-fluorophore labeling for targeted drug delivery (Kang et al., 2010). This system was conjugated with a sgc8 aptamer that showed high binding and internalization ability for targeted CEM cells. The flow cytometry and confocal imaging experiments showed sgc8-modified liposomes could deliver loaded drug to targeted cancer cells with high specificity and excellent efficiency. In recent attempts, the CRISPR/Cas9 complex were packaged into aptamer-functionalized liposomes for specific cancer gene therapy (Gong et al., 2020). For example, Liang *et al.* developed an aptamer-based lipopolymer for tumor-specific delivery of CRISPR/Cas9 to regulate VEGFA in osteosarcoma (Liang et al., 2017). In this system, LC09 aptamer could facilitate the selective distribution of CRISPR/Cas9 plasmids to decrease VEGFA expression, leading to inhibite orthotopic osteosarcoma malignancy and lung metastasis.

Another promising type of aptamer-based organic nanomaterial is the micelle structure. This drug delivery system displays excellent binding ability of aptamers to target due to the multivalent effect. Thus, it can be developed for numerous bioapplications (Wu et al., 2010). In 2018, Li *et al.* developed a cross-linked aptamer-lipid micelle system for excellent stability and specificity in target-cell recognition (Li et al., 2018a). In this facile approach, aptamer and lipid segments were linked to a methacrylamide branch via an efficient photoinduced polymerization process. In contrast to traditional aptamer-lipid micelles, this reported system provided better biostability in a cellular environment, further improving the targeting ability for imaging applications. In another fashion study, a novel aptamer-prodrug conjugate micelle was prepared by combining hydrophobic prodrug bases and bioorthogonal chemistry for hydrogen peroxide and pH-independent cancer chemodynamic therapy (Xuan et al., 2020). In depth mechanistic work reveal that, this system could be activated by intracellular Fe^2+^ to generate toxic C-centered free radicals self-circularly via cascading bioorthogonal reactions.

Among aptamer-based organic nanomaterial systems, target-responsive DNA hydrogels exhibited superior mechanical properties and programmable features and were widely used in biomedical and pharmaceutical applications (Li et al., 2016). In 2008, the first adenosine-responsive hydrogel was developed for potential drug release. In this work, two oligonucleotide-incorporated polyacrylamide and rationally designed cross-linking oligonucleotides were used to form the DNA nanohydrogels. The DNA linker contained the aptamer sequence for adenosine. When existing adenosine molecules, the aptamer will bind to target molecules, resulting in the breakdown of the cross-links and the dissolution of the hydrogel. Thus, this system could be explored for target-responsive drug release (Yang et al., 2008). In other elegant example, Yao *et al.* reported a physically cross-linked DNA network to fish bone marrow mesenchymal stem cells (BMSCs) from numerous nontarget cells (Yao et al., 2020). This nanomaterial containing a Apt19S aptamer sequence provided a 3D microenvironment to maintain excellent activity of captured stem cells.

In addition, due to the principle of complementary base pairing, aptamers can be easily integrated to prepare various DNA nanostructures for specific cancer cell recognition and subsequent applications (Seeman and Sleiman, 2018). As an important DNA-based nanostructures, DNA origami have been modified with various small molecule drugs, functional NA sequences, and nanomaterials (Bolaños Quiñones et al., 2018). In 2018, a smart DNA nanorobot was reported by Li’s group for intelligent drug delivery in cancer therapy. Because of functionalizing on the outside with aptamer AS1411, this DNA nanorobot can specifaically deliver thrombin to tumor-associated blood vessels for inhibiting the tumor growth (Li et al., 2018b). Subsquently, the aptamer-functionalized DNA Origami, named Apt-Dox-orgami-ASO, was developed by *Pan*’s group to co-deliver Dox and antisense oligonucleotides (ASOs) in cancer cells. This multifunctional DNA origami-based nanocarrier was precisely synthesized to adsorb Dox and load Bcl2 and P-gp ASOs for the efficient therapy of drug-resistant cancer (Pan et al., 2020). As cargo carriers, all kinds of aptamer-based DNA nanostructures have been explored By molecular engineering for targeted drug delivery in cancer therapy (Hu et al., 2018).

## 5 Conclusion

In the past decades, the achieved developments proved that aptamers had broad potential in the research field of cancer therapy. Multiple unique properties of aptamers attracted considerable attention in the development of aptamers as nucleic acids-functionalized alternatives to folic acid, peptides and antibodies for targeted drug delivery. This short review summarizes some recent advances of aptamer-based systems in cancer therapy. In fact, the exploration of aptamers and aptamer-drug conjugates is still in a relatively early stage. Considerable efforts should be made to overcome their bottlenecks in the clinical application. In addition, the most remarkable achievements of aptamers involved their combination with nanomaterials, enhancing the specificity of the diagnostic signal and leading to excellent target cancer cell recognition and delivery. In summary, the above description showed the versatility and therapeutic applicability of aptamers. However, multiple challenges, including poor biostability, short half-lives *in vivo* and unclear mechanism of endosomal escape and drug release, need to be overcome before moving forward to clinical application. Furthermore, more systematic research on organ toxicity, the safety on genomics and proteomics, the large-scale production technology and costs need to be further investigated. Despite these limitations, the rapid development of chemistry and materials encourages us to explore aptamer-based drug delivery systems with high therapeutic effects.
